# Detection of genome-wide copy number variations in two chicken lines divergently selected for abdominal fat content

**DOI:** 10.1186/1471-2164-15-517

**Published:** 2014-06-24

**Authors:** Hui Zhang, Zhi-Qiang Du, Jia-Qiang Dong, Hai-Xia Wang, Hong-Yan Shi, Ning Wang, Shou-Zhi Wang, Hui Li

**Affiliations:** Key Laboratory of Chicken Genetics and Breeding, Ministry of Agriculture, Harbin, 150030 P.R China; Key Laboratory of Animal Genetics, Breeding and Reproduction, Education Department of Heilongjiang Province, Harbin, 150030 P.R China; College of Animal Science and Technology, Northeast Agricultural University, Harbin, 150030 P.R China

**Keywords:** Chicken, Copy number variation (CNV), Abdominal fat

## Abstract

**Background:**

The chicken (*Gallus gallus*) is an important model organism that bridges the evolutionary gap between mammals and other vertebrates. Copy number variations (CNVs) are a form of genomic structural variation widely distributed in the genome. CNV analysis has recently gained greater attention and momentum, as the identification of CNVs can contribute to a better understanding of traits important to both humans and other animals. To detect chicken CNVs, we genotyped 475 animals derived from two broiler chicken lines divergently selected for abdominal fat content using chicken 60 K SNP array, which is a high-throughput method widely used in chicken genomics studies.

**Results:**

Using PennCNV algorithm, we detected 438 and 291 CNVs in the lean and fat lines, respectively, corresponding to 271 and 188 CNV regions (CNVRs), which were obtained by merging overlapping CNVs. Out of these CNVRs, 99% were confirmed also by the CNVPartition program. These CNVRs covered 40.26 and 30.60 Mb of the chicken genome in the lean and fat lines, respectively. Moreover, CNVRs included 176 loss, 68 gain and 27 both (i.e. loss and gain within the same region) events in the lean line, and 143 loss, 25 gain and 20 both events in the fat line. Ten CNVRs were chosen for the validation experiment using qPCR method, and all of them were confirmed in at least one qPCR assay. We found a total of 886 genes located within these CNVRs, and Gene Ontology (GO) and Kyoto Encyclopedia of Genes and Genomes (KEGG) pathway analyses showed they could play various roles in a number of biological processes. Integrating the results of CNVRs, known quantitative trait loci (QTL) and selective sweeps for abdominal fat content suggested that some genes (including *SLC9A3*, *GNAL*, *SPOCK3*, *ANXA10*, *HELIOS*, *MYLK*, *CCDC14*, *SPAG9*, *SOX5*, *VSNL1*, *SMC6*, *GEN1*, *MSGN1* and *ZPAX*) may be important for abdominal fat deposition in the chicken.

**Conclusions:**

Our study provided a genome-wide CNVR map of the chicken genome, thereby contributing to our understanding of genomic structural variations and their potential roles in abdominal fat content in the chicken.

**Electronic supplementary material:**

The online version of this article (doi:10.1186/1471-2164-15-517) contains supplementary material, which is available to authorized users.

## Background

Recently, genome-wide association studies (GWAS) have been successfully used in uncovering key genes or markers associated with complex diseases in humans and economically important traits in domestic animals [1, 2]. Based on genotyping data collected from high-throughput SNP chips, new genomic structural variations have been found in the human genome [3]. Copy number variations (CNVs) are a form of genomic structural variation, defined by DNA segments ranging from kilobases (kb) to megabses (Mb) in size, exhibiting differences in copy numbers when comparing two or more genomes [3, 4]. This type of variation includes submicroscopic insertions, deletions and segmental duplications, as well as inversions and translocations [4–6].

CNV detection methods include the use of comparative genomic hybridization (CGH) arrays, SNP arrays, and next-generation sequencing (NGS), and efficient algorithms and softwares are developed to analyze the generated large-scale data. SNP array genotyping offers a number of advantages, low cost, dense coverage and high throughput, therefore, many studies have focused on designing efficient algorithms and softwares to detect reliable CNVs using SNP array data [7, 8], including CNVPartition (http://www.illumina.com), QuantiSNP [9], PennCNV [10], Birdsuite [11], Cokgen [12], Gada [13], and CONAN [14]. These programs have their own strengths and weaknesses [15, 16]. PennCNV distinguishes itself from other algorithms by incorporating multiple information sources (allele frequency, signal intensity, allelic intensity ratio, and distances between SNPs), and by fitting regression models with GC content, it can also overcome the issue of “genomic waves” [17, 18].

CNV can affect gene expression levels, since it contains or disrupts multiple gene coding regions or regulatory elements, which could then lead to phenotypic variation [19]. Not only have CNV studies been performed in humans [19–21], but also in domestic animals, including dogs [22–26], cattle [27–31], swine [32–36], sheep [37–39] and goats [40, 41]. In humans, CNV studies focus mainly on disease development [20], and a multi-allelic CNV encompassing the salivary amylase gene (*AMY1*) was found to be significantly associated with body mass index (BMI) and obesity [21]. In dogs, detection of CNVs responsible for infertility found two genomic regions harboring two important genes for spermatogenesis, *DNM2* and *TEKT1* [26]. However, in domestic animals, most studies were limited on the identification of CNVs and the construction of CNV maps [27–31, 35].

Chicken (*Gallus gallus*) is a classical avian model, and an economically important farm animal, too. The chicken is the first livestock species to have its genome sequenced, and a large number of SNPs have been identified since then. Besides these SNPs, other genomic structural variations are also detected in the chicken genome, such as CNVs. The pea-comb phenotype is caused by a CNV in intron 1 of *SOX5* on chicken chromosome 1 (GGA1) [42]; the late feathering locus includes a partial duplication of the *PRLR* and *SPEF2* genes on GGAZ [43]; and the dark brown plumage color on GGA1 [44] and the dermal hyperpigmentation on GGA20 [45] are also associated with CNVs. Additional large number of CNVs had been detected in the chicken using CGH array, SNP chip and genome sequencing. Using CGH arrays, Wang *et al.* examined ten birds and identified 96 CNVs corresponding to approximately 1.3% of the chicken genome [46]; another study detected 130 CNVRs in four chicken breeds (Cobb broiler, White Leghorn, Chinese Dou and Chinese Dehong) [47]; and Crooijmans *et al.* detected 3,154 CNVs, grouped into 1,556 CNVRs in a variety of chicken breeds [48]. Using the SNP chip, Jia *et al.* identified 209 CNVRs in two distinct chicken lines (White Leghorn and dwarf) [49]. Using the sequencing method, Fan *et al.* identified 8,839 CNVs in two domestic chickens (Silkie and the Taiwanese native chicken L2) [50]. Two other papers reported several CNVs putatively associated with chicken diseases [51, 52]. As in other domestic animals, CNV studies in chickens are also limited in their power. Majority of CNVs detected are relatively large in size, of low resolution, and could contain a high amount of false positives. Genome-wide analysis of CNVs in chicken populations from different genetic backgrounds could help validate CNV regions detected in various studies.

In the present study, the chicken 60 k SNP chip was used to perform genome-wide CNV detection in a population of 475 birds from two broiler lines divergently selected for abdominal fat content (lean and fat lines) [53]. The two lines were selected for more than ten generations and abdominal fat percentage of the lean and fat lines was significantly different from the 4th generation and onwards. We used two methods, PennCNV and CNVPartition, to carry out CNV analysis. Our study provided a comprehensive map of CNVs, which is helpful in understanding genomic variation in the chicken genome, validating CNVs detected in previous studies, and providing preliminary data for investigating the association between CNVs and various phenotypes of economical importance, e.g. abdominal fat content.

## Methods

### Ethics statement

All animal work was conducted according to the guidelines for the Care and Use of Experimental Animals established by the Ministry of Science and Technology of the People’s Republic of China (approval number: 2006–398), and approved by the Laboratory Animal Management Committee of Northeast Agricultural University.

### Animals

In total, 475 birds (203 and 272 individuals from the lean and fat lines, respectively) from the 11th generation population of Northeast Agricultural University broiler lines divergently selected for abdominal fat content (NEAUHLF) were used. Detailed information regarding NEAUHLF has been published previously [53]. Briefly, after 11 generations of divergent selection for abdominal fatness, the abdominal fat percentage of the fat broiler line at 7 weeks of age was 3.59 times more than that of the lean line. All birds were kept in similar environmental conditions and had free access to feed and water.

### Genotyping and quality control

Genomic DNA samples were extracted from blood using a standard phenol/chloroform method, and DNA sample quality was determined using spectrophotometry and agarose gel electrophoresis. The Illumina chicken 60 k SNP chip [54] containing 57,636 SNPs was used, and genotyping data were generated using BeadStudio (Version 3.2.2). Quality control was performed using the following default cutoffs: LRR standard deviation, 0.30; BAF drift, 0.01; and waviness factor, 0.05 [47]. After having removed SNPs of low quality, a total of 475 birds, and 48,035 SNPs on 28 autosomes and the Z sex chromosome were kept for CNV detection.

### Identification of chicken CNVs using PennCNV

PennCNV software [33] was used to identify chicken CNVs. The PennCNV algorithm incorporates multiple information sources, including LRR and BAF of each SNP marker, and the population frequency of B allele (PFB). Both LRR and BAF were exported from BeadStudio using default clustering files for each SNP. PFB was calculated based on the BAF of each marker. Furthermore, PennCNV integrates a computational approach, by fitting regression models with GC content to overcome the issue of “genomic waves”. Chicken gcmodel files were generated by calculating the GC content of 1 Mb genomic regions surrounding each marker (500 kb on each side), and genomic waves were then adjusted using the option -gcmodel. CNV was chosen based on two criteria: first, it must contain three or more consecutive SNPs, and second, it must be present in at least one animal. CNV regions (CNVRs) were determined by combining overlapping CNVs.

### CNV calling using CNVPartition

The CNVPartition software [55] was employed to analyze the same data set, with the confidence score threshold set at 35, in order to verify CNVs detected by PennCNV. In addition, the CNVRs detected by both CNVPartition and PennCNV were cross-checked.

### Validation of CNVR by qPCR

The quantitative real-time PCR (qPCR) method was used to validate ten CNVRs identified by both PennCNV and CNVPartition. For each of the ten CNVRs, we selected animals predicted by PennCNV to have different status of CNVs (loss, gain or both) for the validation experiment. Together with three other birds predicted by PennCNV to be normal, a total of 65 birds were used. All qPCR experiments were conducted on an ABI Prism 7500 sequence detection system (Applied Biosystems), using SYBR green chemistry in three replicates. Each reaction was performed with a volume of 10 μl. Primers were designed using Primer premier 5.0 and Oligo 6.0, by limiting product sizes in a range from 100 to 200 bp (Additional file 1: Table S1). The vimentin (*VIM*) gene, with a single copy in the chicken genome, was chosen as the reference [56]. For every CNVR, three samples without CNVRs were used as negative controls. The condition for thermal cycle was as follows: 2 min at 50°C, 10 min at 95°C and 40 cycles of 15 s at 95°C and 1 min at 60°C. The 2^-ΔΔCt^ method was used to calculate the copy numbers [57].

### Gene detection and functional annotation

Genes located in the identified CNVRs were retrieved from Ensembl (http://www.ensembl.org) (Galgal4). Functional annotation of genes was performed using DAVID bioinformatics resources 6.7 (http://david.abcc.ncifcrf.gov/summary.jsp) [58] for Gene Ontology (GO) terms [59] and Kyoto Encyclopedia of Genes and Genomes (KEGG) [60] pathway analysis. Statistical significance was determined using a *P*-value < 0.05.

## Results

### Genome-wide identification of CNVs

**Figure 1:**
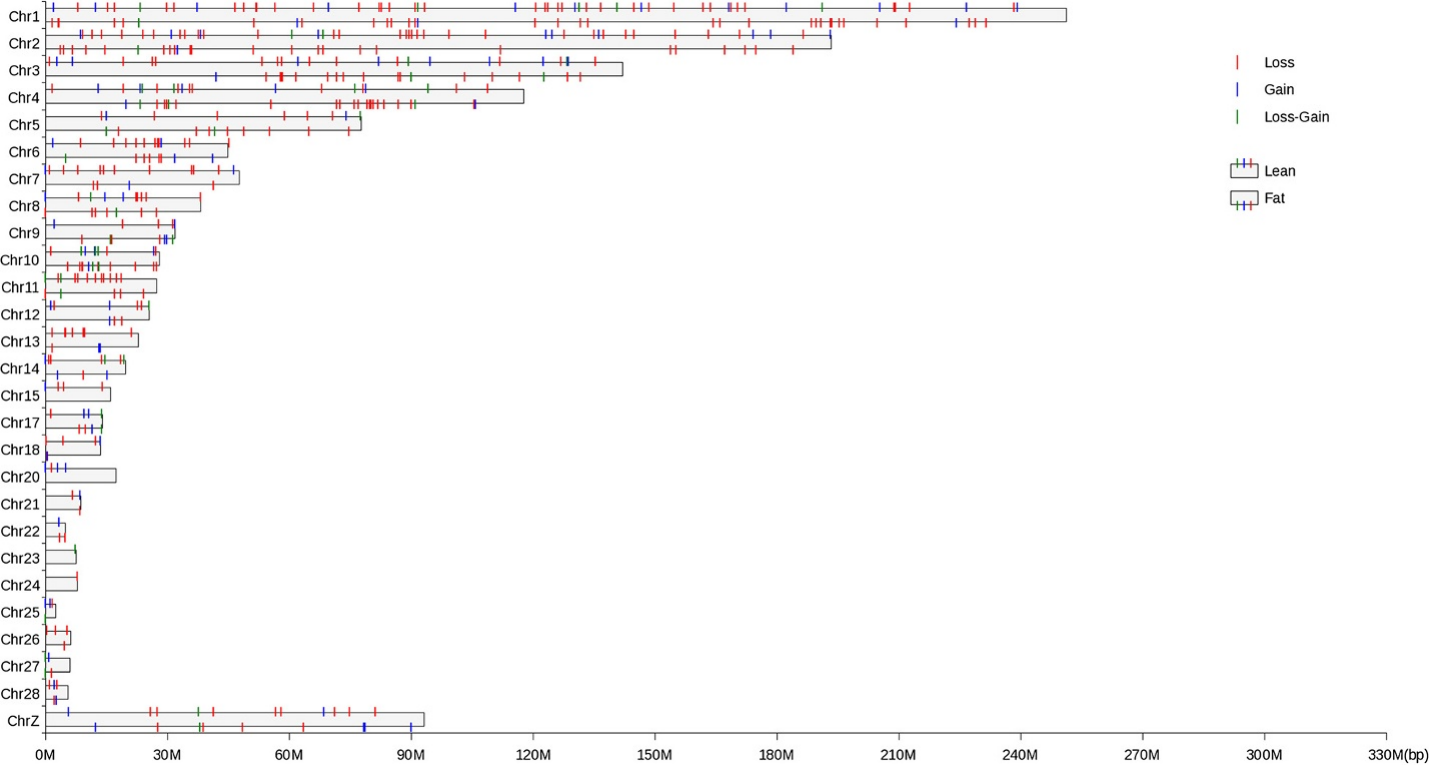
**Distribution of CNVRs identified in the lean and fat lines.**

Using 28 autosomal chromosome and the Z sex chromosome, PennCNV identified 438 and 291 CNVs in the lean and fat lines, respectively. Among these CNVs, 17 were common to both lean and fat lines. Combining overlapping CNVs, we identified a total of 271 and 188 CNVRs across the whole genome, covering 40.26 and 30.60 Mb (3.92% and 2.98% of whole genome length) in the lean and fat lines, respectively (Figure 1). Among CNVRs in the lean line, there were 177 loss, 68 gain and 27 both (i.e. loss and gain within the same region) events, while in the fat line, there were 143 loss, 25 gain and 20 both events. In the lean line, the length of CNVRs ranged from 6.23 to 932.14 kb with a mean of 148.77 kb and median of 107.81 kb. In the fat line, CNVRs ranged from 0.33 to 1442.99 kb, with a mean of 163.43 kb and median of 99.81 kb. CNVR locations and characteristics across all 28 autosomal and the Z sex chromosomes were summarized (Figure 1, Additional file 2: Table S2, Additional file 3: Table S3). It is apparent that CNVRs are not uniformly distributed across different chromosomes. No CNVRs were detected on chromosomes 16 and 19 in the lean line, and on chromosomes 15, 16, 19, 20, 23 and 24 in the fat line. Chromosome 1 harbored the largest number of CNVRs, with 53 and 35 in the lean and fat lines, respectively.

The CNVPartition program implemented in Illumina GenomeStudio software was also used to verify the CNVRs detected by PennCNV. There were only 4 and 3 CNVRs, out of 271 and 188 CNVRs in the lean and fat lines respectively, missed by CNVPartition (Additional file 2: Table S2, Additional file 3: Table S3). When compared the results obtained from the PennCNV and CNVPartition programs, we saw that most of the CNVRs identified by the CNVPartition were larger in size, and can contain multiple CNVRs identified by PennCNV.

### CNV validation by quantitative PCR

**Figure 2:**
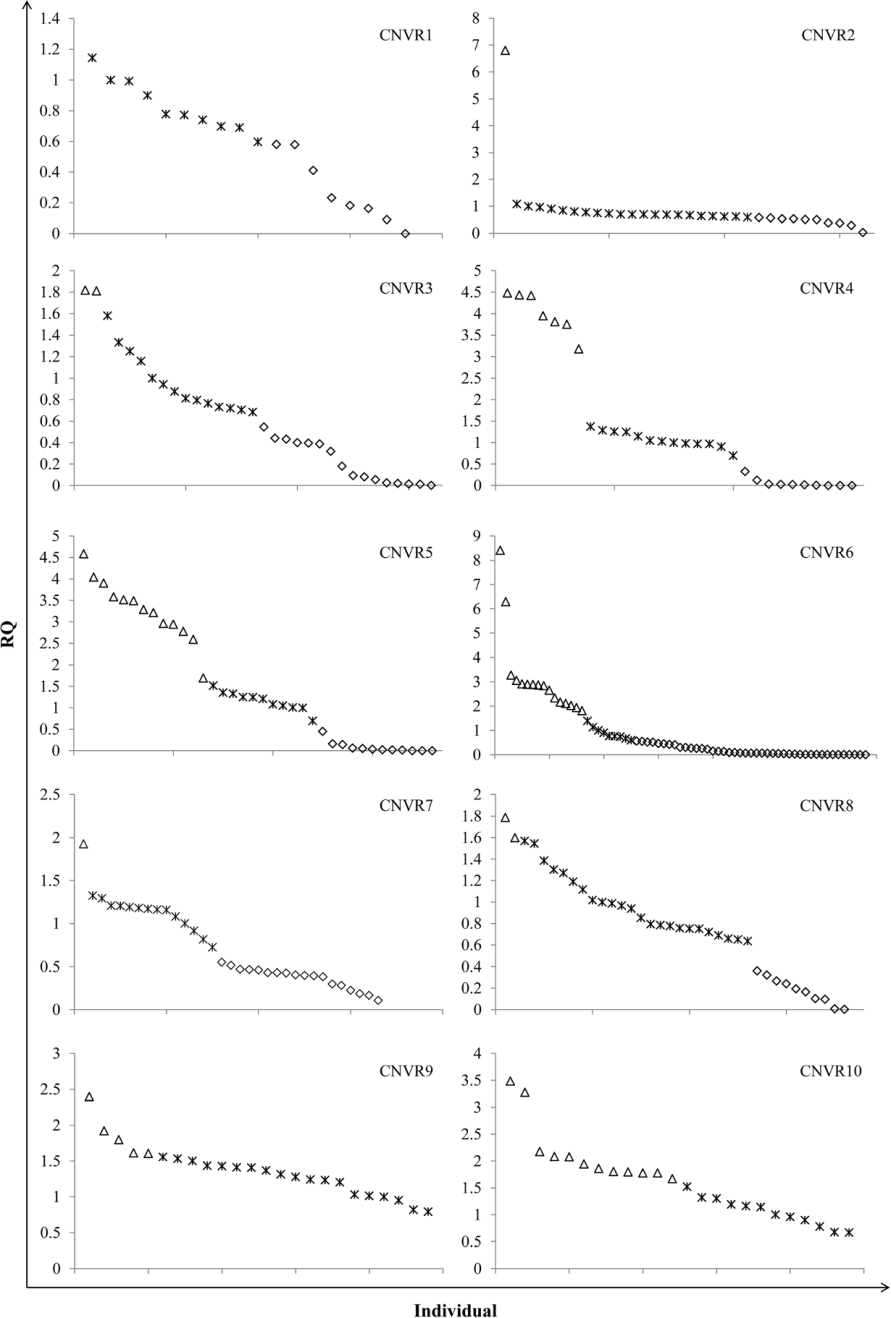
**Verification of 10 detected CNVRs by qPCR.** CNVR1-10 represents 10 CNVRs selected for qPCR validation. X-axis shows the individuals used in the validation experiment. Y-axis shows the relative quantification (RQ) values obtained by qPCR. Different shapes denote samples with different RQ values. Asterisk: samples with RQ values about 1 denote normal individuals (two copies); Box: samples with RQ values below 0.59 (ln^1.5^) denote copy number loss individuals; Triangle: samples with RQ values about 1.59 (ln^3^) or more denote copy number gain individuals (>three copies) [61].

The quantitative real-time PCR experiments were performed to validate the CNVRs detected in the current study. Ten putative CNVRs were selected, and represented different status of copy numbers (predicted to be from gain, loss, or both events) (Table 1). We performed 333 qPCR assays in 65 animals. Out of the 333 qPCR assays, 204 (61%) were in agreement with the CNV prediction made by PennCNV. Direct counting of the CNVRs confirmed that all 10 CNVRs had copy number variations in at least one qPCR assay (Figure 2, Table 1).

**Table 1:** **QPCR validation results**

CNVR no.	Position	Validated	Validated type	Detected type
CNVR1	chr1: 68169551-68304034	Yes	Loss	Loss
CNVR2	chr1: 18444585-19186283	Yes	Loss and gain	Loss and gain
CNVR3	chrZ: 62739744-62787579	Yes	Loss and gain	Gain
CNVR4	chr5: 33373065-33628432	Yes	Loss and gain	Loss and gain
CNVR5	chrZ: 9933957-10124452	Yes	Loss and gain	Gain
CNVR6	chr17: 11092462-11179187	Yes	Loss and gain	Loss and gain
CNVR7	chr2: 149276922-149301687	Yes	Loss and gain	Loss
CNVR8	chr11: 3196613-3453273	Yes	Loss and gain	Loss and gain
CNVR9	chr10: 7945101-7980528	Yes	Gain	Gain
CNVR10	chr12: 1112437-1143728	Yes	Gain	Gain

### Genes located in CNVRs

Within the 271 CNVRs identified in the lean line, 204 (75.3%) CNVRs contained 626 genes retrieved from Ensembl, and the remaining 67 CNVRs did not contain any annotated genes. Within the 188 CNVRs identified in the fat line, 140 (74.5%) CNVRs had 374 genes, and no annotated genes located in the other 48 CNVRs, either. There were 114 common genes detected in both chicken lines. Therefore, a total of 886 genes were identified within the detected CNVRs.

Functional enrichment analyses found that a total of 1744 GO terms for the 886 genes, with eight GO terms statistically significant (*P* < 0.05) (Table 2). Significant GO terms were mainly involved in interleukin-1 binding, interleukin-1 receptor activity, hydrogen antiporter activity, calcium ion binding, solute antiporter activity, plasma membrane, antiporter activity, and cation antiporter activity. GO terms with marginal significance were mainly involved in cell-cell adhesion, cytokine receptor activity, dystrophin-associated glycoprotein complex, homophilic cell adhesion and hydrogen antiporter activity (monovalent cation and sodium). KEGG pathway analyses showed that genes in CNVRs were enriched in 95 pathways, two of them statistically significant (calcium signaling and N-Glycan biosynthesis pathways) (Table 2).

**Table 2:** **Significant GO categories and KEGG pathways associated with genes in CNVRs**

Category	Term	Description	P-value
GOTERM_MF_FAT	GO:0019966	Interleukin-1 binding	0.00
GOTERM_MF_FAT	GO:0004908	Interleukin-1 receptor activity	0.00
GOTERM_MF_FAT	GO:0015299	Solute: hydrogenantiporter activity	0.02
GOTERM_MF_FAT	GO:0005509	Calcium ion binding	0.02
GOTERM_MF_FAT	GO:0015300	Solute: soluteantiporter activity	0.02
GOTERM_CC_FAT	GO:0005886	Plasma membrane	0.03
GOTERM_MF_FAT	GO:0015297	Antiporter activity	0.03
GOTERM_MF_FAT	GO:0015298	Solute: cationantiporter activity	0.03
KEGG_PATHWAY	gga04020	Calcium signaling pathway	0.02
KEGG_PATHWAY	gga00510	N-Glycan biosynthesis	0.03

### Integrative analysis of CNVRs, QTLs, and selective sweeps for abdominal fat content

In the chicken QTL database (http://www.animalgenome.org/cgi-bin/QTLdb/GG/index; May 21, 2013), there were 3,808 quantitative trait loci (QTLs) affecting 296 traits, including 291 QTLs for abdominal fat weight and percentage. In the lean line, 160 CNVRs overlapped with 74 QTLs affecting abdominal fat weight or percentage. Furthermore, for the 271 CNVRs in the current study and 5357 core regions detected in the previous seletive sweep study [62], we found that 225 CNVRs overlapped with 311 core regions, after comparing the CNVR and selective sweep results. In the 311 core regions, 10 regions reached statistical significance of *P* < 0.01. These 10 core regions related to 10 CNVRs, with six CNVRs located in QTL regions for abdominal fat content. Eight genes were found within these six CNVRs, including solute carrier family 9 member 3 (*SLC9A3*), guanine nucleotide binding protein (G-protein), alpha activating activity polypeptide, olfactory type (*GNAL*), sparc/osteonectin, cwcv and kazal-like domains proteoglycan (testican) 3 (*SPOCK3*), annexin A10 (*ANXA10*), IKAROS family zinc finger 2 (*HELIOS*), myosin light chain kinase (*MYLK*), coiled-coil domain containing 14 (*CCDC14*), and sperm-associated antigen 9 (*SPAG9*).

In the fat line, 112 CNVRs overlapped with 66 QTLs affecting abdominal fat weight or percentage in the chicken QTL database. In addition, for the 188 CNVRs in the current study and 5593 core regions detected in the previous seletive sweep study [62], we found that 140 CNVRs overlapped with 203 core regions, and in the 203 core regions, 6 regions reached statistical significance of *P* < 0.01. These 6 core regions related to six CNVRs, with four CNVRs located in abdominal fat QTLs. Six genes were found within these four CNVRs, including SRY (sex determining region Y)-box 5 (*SOX5*), visinin-like 1 (*VSNL1*), structural maintenance of chromosomes 6 (*SMC6*), GEN endonuclease homolog 1 (*GEN1*), mesogenin 1 (*MSGN1*) and zona pellucida protein (*ZPAX*).

## Discussion

The chicken 60 k SNP array was originally developed for high-throughput SNP genotyping in GWAS studies. Although CNV detection is feasible with this SNP panel, it is of less power due to low marker density, non-uniform SNP distribution along chicken chromosomes, and a lack of non-polymorphic probes specifically designed for CNV identification [63]. Thus, using this array, typically only large CNVRs could be identified.

Several algorithms have been developed to identify CNVs, including CNVPartition, QuantiSNP, PennCNV, Birdsuite, Cokgen, Gada, and CONAN [9–14]. Each algorithm has its own strength and weakness [15, 16]. In the present study, the PennCNV algorithm was used for CNV detection, and CNVPartition was employed to verify the CNVs detected by PennCNV. We found that 99% of the CNVRs detected by PennCNV could be verified by the CNVPartition program. This high ratio indicated that the CNVRs detected in this study were credible and the following discussion was based on the PennCNV results.

We used two lines divergently selected for abdominal fat content to detect CNVs in the chicken, and found the lean line had more CNVs than the fat line (438 *vs* 291). One of the reason could be due to different number of animals (203 and 272 individuals in the lean and fat lines, respectively). Additionally, these two lines have different selection signatures as reported previously [62], which suggests that artificial selection for abdominal fat could also lead to CNV alterations between these two lines.

We compared our results with several previous reports on chicken CNVs. The first study was reported by Griffin *et al.* [64]. They used the CGH array and detected 12 CNVs in broiler and layer genomes, compared with the Red Jungle Fowl. Two of these 12 CNVs overlapped with our results (Additional file 4: Table S4). Wang *et al.* detected 96 CNVs in three chicken lines (Cornish Rock broiler, Leghorn, and Rhode Island Red) using whole-genome tiling arrays [46]. Of these 96 CNVs, 14 CNVs overlapped with our results (Additional file 4: Table S4). In 2012, Wang *et al.* detected 130 CNVRs in four chicken breeds (Cobb broiler, White Leghorn, Chinese Dou and Chinese Dehong) using CGH arrays, with 16 overlapping CNVs (Additional file 4: Table S4) [47]. In the same year, Jia *et al.* identified 209 CNVRs in two distinct chicken lines (White Leghorn and dwarf) using chicken 60 k SNP arrays, with 47 overlapping CNVRs (Additional file 4: Table S4) [49]. Luo *et al.* identified 45 CNVs in four chicken lines (L6_3_, L7_2_, RCS-L, and RCS-M), with two CNVs overlapping with our CNVRs [51]. Crooijmans *et al.* detected 1556 CNVRs using the CGH arrays in a wide variety of chicken breeds, with 140 overlapping CNVRs with our current study [48]. In total, 181 of 459 CNVRs (271 and 188 CNVRs in lean and fat lines, respectively) (39%) detected in our study were also detected in previous studies (Additional file 4: Table S4). Potential reasons for the observed differences include the following three considerations. Firstly, the populations are of different sizes and genetic background; Secondly, different array platforms are used, either SNP genotyping or CGH arrays; Thirdly, genomic waves can interfere with accurate CNV detection [41, 65]. Genomic waves refer to signal intensity patterns across all chromosomes, with different samples showing highly variable magnitudes of waviness [41]. In our study, we adjusted for genomic waves using the -gcmodel option in PennCNV. Genomic waves were generally not considered in other studies. Apart from low overlapping rates between different chicken CNV studies, the same issue was also encountered in other animals [65–67].

In previous observations, CNVs are preferentially located in gene-poor regions [68, 69]. It is speculated that CNVs present in gene-rich regions may be deleterious and under purifying selection [70]. In the chicken genome, there are approximately 28,000 genes (data from the GeneChip®Chicken Genome Array Profile), and 886 (3.16%) annotated genes located in the 271 and 188 CNVRs in the lean and fat lines, respectively, were identified in our current study. These CNVRs covered 3.92 and 2.98% of the chicken genome in the lean and fat lines, respectively. Therefore, we can not state that these CNVRs locate in gene-poor or gene-rich regions.

QPCR is often used to validate novel CNVRs, but confirmation rates are usually not very high [11, 20, 21]. For instance, Fadista *et al.* [20] and Hou *et al.* [65] confirmed 50 and 60% of CNVRs selected for validation, respectively. Our validation rate was 61% (204 out of the 333 qPCR assays), comparable to the results of other studies.

Comparing CNVs detected in our current study with known QTLs (in the QTL database) and selective sweeps for abdominal fat content [62], we identified 14 genes (8 and 6 in the lean and fat lines, respectively). For the eight genes in the lean line, we found *SLC9A3*, *GNAL*, *ANXA10*, *MYLK*, *CCDC14*, and *SPAG9* expressed in chicken pre-adipocytes, and *SLC9A3*, *GNAL*, *ANXA10*, *HELIOS*, *MYLK*, *CCDC14*, and *SPAG9* expressed in both chicken abdominal fat and liver tissues (data not published). For the six genes in the fat line, we found *SOX5*, *VSNL1*, *SMC6*, and *GEN1* expressed in chicken pre-adipocytes, *GEN1*, *SMC6*, *SOX5*, and *VSNL1* expressed in chicken abdominal fat tissue, and *SOX5*, *VSNL1*, and *SMC6* expressed in chicken liver tissue (data not published). Basic functions of these 14 genes are described as follows.

*SLC9A3* is also known as sodium–hydrogen antiporter 3, or sodium–hydrogen exchanger 3 (*NHE3*) [71]. *SLC9A3* is expressed in human intestine, stomach, respiratory tract, kidneys, glandular and epithelial cells [72]. *SLC9A3* is present in the brush-border of intestinal Na + -absorptive cells and renal proximal tubules, playing an important role in gastrointestinal and renal Na + absorption [73], and suggesting it may be involved in food digestion and nutrient absorption, and in turn, abdominal fat deposition.

G-proteins are divided into four subfamilies according to their α-subunits (Gαs, Gαi/o, Gαq, and Gα12) [74]. Gα subunits interact with both receptor and effect or molecules, and are considered the functional component of G-proteins. *GNAL* shares 88% amino acid homology with Gαs, and is considered a member of the Gαs family [74]. Although *GNAL* was originally discovered in olfactory neuroepithelium and striatum, it is also present in pancreatic β-cells, testis, spleen, lung, and heart [75]. In addition, this gene is highly expressed in adipose tissue (http://www.genatlas.org/), indicating it may be associated with abdominal fat deposition.

*SPOCK3* encodes a member of the novel family of calcium-binding proteoglycan proteins that contain thyroglobulin type-1 and Kazal-like domains. Encoded SPOCK3 protein may play a key role in adult T-cell leukemia by inhibiting membrane-type matrix metalloproteinase activity [76]. *SPOCK3* is expressed in the mouse nervous system [77].

*ANXA10* belongs to the annexin family, and is over-expressed in oral squamous cell carcinoma-derived cell lines [78]. *ANXA10* plays an important role in cellular functioning of endocytosis and exocytosis, anticoagulant activity, cytoskeletal interactions, differentiation, and cellular proliferation [79, 80]. Moreover, *ANXA10* shows relevant malignancy in Barrett’s esophagus, gastric cancer, and bladder cancer [81–83]. *ANXA10* is expressed in the digestive system including liver and stomach tissues (http://www.genatlas.org/), indicating it may affect food digestion and absorption, and consequently be associated with fat deposition.

*Helios* is a member of the Ikaros transcription factor family, and preferentially expressed by regulatory T cells [84]. Previous work has shown that obese patients with insulin resistance have decreased *HELIOS* but increased *FOXP3* mRNA expression in visceral adipose tissue [85]. *Helios* is expressed in ectodermal and neuroectodermal-derived tissues [86].

*MYLK* is a muscle member of the immunoglobulin gene superfamily, and encodes myosin light chain kinase, a calcium/calmodulin dependent enzyme. Genetic and functional studies show that heterozygous loss-of-function mutations in *MYLK* are associated with aortic dissection [87]. *MYLK* is highly expressed in heart, prostate, trachea tissues, and the digestive system (including esophagus and small intestine), suggesting this gene is involved in food digestion and absorption, and consequently associated with fat deposition.

*CCDC14* is a protein-coding gene with unknown function. *CCDC14* is expressed in male testis tissue (http://www.genatlas.org/).

*SPAG9* is a novel member of c-Jun NH2 -terminal kinase (*JNK*) interacting proteins, exclusively expressed in testis [88]. *SPAG9* may play a key role in reproductive processes, and tumor growth and development [88, 89].

*SOX5* is a member of the SOX (SRY-related HMG-box) family, and involved in regulation of embryonic development and cell fate determination [90]. In chicken, CNV in intron 1 of *SOX5* can cause the Pea-comb phenotype [42]. *SOX5* is expressed in brain, spinal cord, testis, lung, and kidney, and can control cell cycle progression in neural progenitors by interfering with the WNT-beta-catenin pathway [91]. A recent study indicated *SOX5* may play an important role in left ventricular mass regulation, a disease that may be affected by abdominal obesity [92].

*VSNL1* is a member of the visinin/recoverin subfamily of neuronal calcium sensor proteins, and highly expressed in human heart and brain [93, 94]. Previous results suggest *VSNL1* regulates heart natriuretic peptide receptor B [93]. The *VSNL1* gene also plays a critical role in regulating cell adhesion and migration via downregulation of fibronectin receptor expression [95]. The *VSNL1* gene is highly expressed in the nervous system.

Structural maintenance of chromosomes (SMC) proteins are a family of related proteins that form the core of three protein complexes. Smc1 and 3 ensure sister chromatids remain associated after DNA replication, as well as playing roles in gene expression and DNA repair [96]. Smc2 and 4 are responsible for chromosome condensation during mitosis [97]. The Smc5-6 complex is required for DNA repair by homologous recombination, although its exact role is not fully understood [98].

*GEN1* is a member of the Rad2/XPG family of monomeric, structure-specific nucleases [99]. This protein family includes N-terminal and internal XPG nuclease motifs, and a helix–hairpin–helix domain [100]. The *GEN1* gene is expressed in pancreas, thymus, brain, testis, lung, and kidney, and has Holliday junction resolvase activity *in vitro*, presumably functioning in homology-driven repair of DNA double-strand breaks [101].

*Msgn1* is a basic helix–loop–helix transcription factor, specifically expressed in the presomitic mesoderm (psm). *Msgn1* controls differentiation and movement of psm progenitor cells, and mouse embryos lacking *Msgn1* exhibit a severely reduced psm and an absence of trunk somites [102, 103].

The vertebrate egg envelope is constructed by a set of related proteins encoded by the zona pellucida (*ZP*) genes [104]. Vertebrate *ZP* genes have six subfamilies: ZPA/ZP2, ZPB/ZP4, ZPC/ZP3, ZP1, ZPAX, and ZPD [105]. The *Zpb* pseudogene was identified in the mouse genome, *Zp1* pseudogene in the dog and bovine genomes, and *Zpax* pseudogene in the human, chimpanzee, macaque, and bovine genomes [105]. *ZP* genes may play an important role in sperm-egg recognition [105].

All these genes are located in QTLs for abdominal fat weight or percentage in the chicken. From the known functions of these genes, *GNAL*, *HELIOS*, and *SOX5* are directly related to adipose tissue metabolism or obesity, while *SLC9A3*, *SPOCK3*, *ANXA10*, *MYLK*, and *VSNL1* may be indirectly related. The function of *CCDC14*, *SPAG9*, *SMC6*, *GEN1*, *MSGN1*, and *ZPAX* on adipose tissue development is unknown. However, further investigation are still needed to examine their functional implications in chicken adipogenesis.

## Conclusions

We have constructed a CNVR map for the broiler chicken using two lines divergently selected for abdominal fat content. In total, 271 and 188 CNVRs in the lean and fat lines were identified, respectively. Integrating detected CNVRs, and results of QTLs and selection signatures for abdominal fat content, 14 genes (including *SLC9A3*, *GNAL*, *SPOCK3*, *ANXA10*, *HELIOS*, *MYLK*, *CCDC14*, *SPAG9*, *SOX5*, *VSNL1*, *SMC6*, *GEN1*, *MSGN1*, and *ZPAX*) were identified as putatively important for chicken abdominal fat content.

### Availability of supporting data

The data sets supporting the results of this article are included within the article and its additional files. The chicken 60 k SNP data presented in this paper have been deposited into Gene Expression Omnibus (http://www.ncbi.nlm.nih.gov/geo/) with the identifier GSE58551.

## Electronic supplementary material

Additional file 1: Table S1: Primers designed for ten CNVRs in quantitative PCR analyses. (DOC 56 KB)

Additional file 2: Table S2: The CNVRs detected in the lean line. (DOC 236 KB)

Additional file 3: Table S3: The CNVRs detected in the fat line. (DOC 174 KB)

Additional file 4: Table S4: Summary of chicken CNVRs identified in the current and previous studies. (XLS 53 KB)

## Abbreviations

AFP: Abdominal fat percentage
CGH: Comparative genomic hybridization
CNV: Copy number variation
CNVR: CNV region
GO: Ontology
GWAS: Genome-wide association studies
kb: Kilobases
KEGG: Kyoto Encyclopedia of Genes and Genomes
Mb: Megabases
NEAUHLF: Northeast Agricultural University broiler lines divergently selected for abdominal fat content
PFB: Population frequency of B allele
SNP: Single nucleotide polymorphism
VLDL: Plasma very low-density lipoprotein.
