# Supplementary material for: Detection of genome-wide copy number variations in two chicken lines divergently selected for abdominal fat content
Source: BMC Genomics. 2014 Jun 24;15:517. doi: 10.1186/1471-2164-15-517 (PMC4092215; doi:10.1186/1471-2164-15-517)
Supplement: Supplementary file 1 — Additional file 1: Table S1: Primers designed for ten CNVRs in quantitative PCR analyses. (DOC 56 KB) [file 12864_2013_6209_MOESM1_ESM.doc]

Additional file 1: Table S1 Primers designed for ten CNVRs in quantitative PCR analyses

| Locus | Primer | Position | Length  (bp) |
| --- | --- | --- | --- |
| VIM | F: GCAGATGCAGTAGGCATTCA  R: GCTGCACTTAGGGCACAAAT | chr2:19730925-19731078 | 154bp |
| CNVR1 | F: CTTCTTTCATTCAGAGATGCC  R: TTCAAACAGCCGAAAGGTAA | chr1:68169477-68169652 | 176bp |
|  | F: CCTGAAGTATTGATTAGGGTAGC  R: ATGTTTATGACCAAGCAGTGAGT | chr1:68185563-68185707 | 145bp |
|  | F: ACTCTGCAAAGGCAAAAC  R: ATCTTCCTGGTGGTCAT | chr1:68213494-68213684 | 191bp |
| CNVR2 | F: TGGTTATTTCAGTAGATTTACACAA  R: AAAGTGCCGTGAAGTTCTGT | chr1:18836477-18836592 | 116bp |
|  | F: ACAAAAAGTTTCTCCTTATTTCAGTCAG  R: AATTTTTCATTGACTTCTACCCTCTTA | chr1:18855379-18855512 | 134bp |
|  | F: CTTGGGTGTCCTGGTAAA  R: AACAGCTCCACATCTTCC | chr1:18898265-18898458 | 194bp |
|  | F: TTTATGTGAATCACAGTATATTTGGATT  R: TAAACCAACTGCCTTTTACCAGA | chr1:18911349-18911519 | 171bp |
| CNVR3 | F: CAGTCCCTCCATTCTTGT  R: TGCTATCGTGGAAGTTGT | chrz:62739683-62739849 | 167bp |
|  | F: TCAGGTGACAATGCAACTAG  R: CTCTTCCCCTTATTGTATTG | chrZ:62762381-6276549 | 169bp |
|  | F: AAAATCACCTCCCTCTGC  R: AGTACCGATGAATGACAAGC | chrZ:62784559-62784662 | 104bp |
|  | F: GTTCATAGTTACAATGTTAATAGCAGCAT  R: GACAAACACGGGTATGTTCAGC | chrz:62787533-62787647 | 115bp |
| CNVR4 | F: AGTGGCACAAGATTCAACCTTTT  R: TAGATTTTGGCATATGGAGCGTA | chrz:33449169-33449325 | 157bp |
|  | F: TAGTTCACAGGTATTAAAATATAGATATAC  R: GGAAAGTGTATTCTTTTAGTATTATTT | chrz:33459375-33459545 | 171bp |
|  | F: TTTCATCTGCCTACTCCC  R: TCCCAGGTTCTGTCTCAC | chr5:33477543-33477696 | 154bp |
| CNVR5 | F: ATTCTTCATTTTCTTTCCTG  R: CTCTGCTACTCAATCTATCATC | chrZ:9975695-9975832 | 138bp |
|  | F: AGAGCCGACAGTAATATGAATGAAG  R: CCAAGTTTAGAGCTAGGAGGGAT | chrz:9995140-9995295 | 156bp |
|  | F: TGCTGTGATGCCTGTGAC  R: TTGCTGCTGACTTACTGTTT | chrz:10005390-10005545 | 156bp |
| CNVR6 | F: AAATAATCAGCGTGTTCCTCAAT  R: GAAGAGAGCAAAAAAAGTCTATGAT | chr17：11148728-11148874 | 147bp |
|  | F: AACTGCAATTCCACCTACTCACA  R: CTTCTCATCCAGCACAGACCTAA | chr17:11157792-11157978 | 187BP |
|  | F: GCCACGTTAGTTCCTTTC  R: CTGTGATTCCGTATGTTATT | chr：11176973-11177162 | 190bp |
|  | F: AGACCTTCTCCAATGCTAGACTTTTACCCCTT  R: GAATGTTGCGCGTGCGCCATC | chr17:11179103-11179286 | 184bp |
| CNVR7 | F: GCTGGGAGACTCTGTGTTATC  R: TGTTCCTGCCTGTATTATTTCT | chr2:149276879-149277023 | 145bp |
|  | F: GCCAATTTCTCATCAGCAAACC  R: AAGACCTTCAAGTGCCTTCCCAT | chr2:149283460-149283592 | 133bp |
|  | F: TGTCCTACTTGTAGTTAAAAATGTTGC  R: GCTTTACCAGTTTGTGGGAGTCT | chr2:149301654-149301813 | 160bp |
| CNVR8 | F: GAATCATGGTAGGAACACTGGAAAT  R: TGTGGGTGCAAAGGACAAGTAC | chr11:3196550-3196687 | 138bp |
|  | F: AGGAACAATGGTATTTGCTTATC  R: ATCCCATTTTCTTCTACAGTATCTC | chr11:3217349-3217541 | 193bp |
|  | F: AGCAATGAAAATGGATAGCAAGC  R: GCAGATTTTAGTGCTTTAATGATAACTT | chr11:3230826-3230968 | 143bp |
|  | F: CCGATGTTTCTGGATTG  R: CCAGCATTCTGAGATTTG | chr11:3238868-3239048 | 181bp |
| CNVR9 | F: CAAAAGCTGTATGGTTTGGAAGA  R: TTAGGCAACACTGTAGAAATGGC | chr10:7945021-7945162 | 142bp |
|  | F: GTGTTGCATGAGGAGATGAGATACC  R: AAAGCATTAGCCCTGGAAAAGC | chr10:7953564-7953728 | 165bp |
|  | F: TTGTAGCCCAGGGTGTTGC  R: AGAGGTCAGTTCACTTGTTCATCG | chr10:7960476-7960617 | 142bp |
| CNVR10 | F: GAAACTTGGTTCCTCTCCAGGCTTG  R: GTTGCCCCGAAATCCAAGGTG | chr12:1112342-1112525 | 184bp |
|  | F: GCTGCTCGTGTCAATGCTGC  R: CCTTGGTACACAGAGTCAGCAGAAC | chr12:1120837-1120969 | 133bp |
|  | F: TAATGCCAGCCTCGGACGG  R: CTGAAATGCAGAACTGACACACGG | chr12:1143655-1143806 | 152bp |
