# Supplementary material for: Detection of genome-wide copy number variations in two chicken lines divergently selected for abdominal fat content
Source: BMC Genomics. 2014 Jun 24;15:517. doi: 10.1186/1471-2164-15-517 (PMC4092215; doi:10.1186/1471-2164-15-517)
Supplement: Supplementary file 2 — Additional file 2: Table S2: The CNVRs detected in the lean line. (DOC 236 KB) [file 12864_2013_6209_MOESM2_ESM.doc]

Additional file 2: Table S2 The CNVRs detected in the lean line

| NO. | Position | Status | Is verified by CNVPartition |
| --- | --- | --- | --- |
| 1 | chr1:1644939-1905345 | Gain | Yes |
| 2 | chr1:6492505-6583362 | Loss | Yes |
| 3 | chr1:9902414-10143550 | Gain | Yes |
| 4 | chr1:12302872-12328332 | Loss | Yes |
| 5 | chr1:13624022-13885922 | Loss | Yes |
| 6 | chr1:18779056-19149987 | Loss and Gain | Yes |
| 7 | chr1:23967005-24299339 | Loss | Yes |
| 8 | chr1:25447345-25474915 | Loss | Yes |
| 9 | chr1:29924920-30030667 | Gain | Yes |
| 10 | chr1:37453758-37622981 | Loss | Yes |
| 11 | chr1:39118012-39288673 | Loss | Yes |
| 12 | chr1:41520639-41553488 | Loss | Yes |
| 13 | chr1:41607141-41684034 | Loss | Yes |
| 14 | chr1:45305010-45745209 | Loss | Yes |
| 15 | chr1:52855688-52940122 | Loss | Yes |
| 16 | chr1:55823609-55944981 | Gain | Yes |
| 17 | chr1:61831294-62109727 | Loss | Yes |
| 18 | chr1:65788155-65818532 | Loss | Yes |
| 19 | chr1:66258202-66503860 | Loss | Yes |
| 20 | chr1:67830818-68213617 | Loss | Yes |
| 21 | chr1:72807948-72967591 | Loss | Yes |
| 22 | chr1:73356229-73632233 | Loss and Gain | Yes |
| 23 | chr1:74753801-75062169 | Loss | Yes |
| 24 | chr1:92658580-92785341 | Gain | Yes |
| 25 | chr1:96557341-96642332 | Loss | Yes |
| 26 | chr1:98463239-98595920 | Loss | Yes |
| 27 | chr1:99058388-99176862 | Loss | Yes |
| 28 | chr1:100994585-101305146 | Loss | Yes |
| 29 | chr1:101849838-102056617 | Loss | Yes |
| 30 | chr1:104313889-104597043 | Gain | Yes |
| 31 | chr1:105192563-105502957 | Loss and Gain | Yes |
| 32 | chr1:106555552-107048406 | Loss | Yes |
| 33 | chr1:109362427-109399702 | Loss | Yes |
| 34 | chr1:112610011-112791888 | Loss and Gain | Yes |
| 35 | chr1:115927107-115959072 | Loss | Yes |
| 36 | chr1:117435846-117602967 | Gain | Yes |
| 37 | chr1:118807490-118878325 | Loss | Yes |
| 38 | chr1:123748267-123838359 | Loss | Yes |
| 39 | chr1:129581080-129788620 | Loss | Yes |
| 40 | chr1:130963080-131137199 | Loss | Yes |
| 41 | chr1:134556500-134591115 | Gain | Yes |
| 42 | chr1:135050327-135114091 | Loss | Yes |
| 43 | chr1:136398212-136580716 | Loss | Yes |
| 44 | chr1:137769174-138298243 | Loss | Yes |
| 45 | chr1:145904334-146209421 | Gain | Yes |
| 46 | chr1:152977566-153253682 | Loss and Gain | Yes |
| 47 | chr1:164290965-164505622 | Gain | Yes |
| 48 | chr1:167083454-167174952 | Loss | Yes |
| 49 | chr1:167422705-167617281 | Loss | Yes |
| 50 | chr1:170159714-170246386 | Loss | Yes |
| 51 | chr1:181454454-181594142 | Gain | Yes |
| 52 | chr1:190787586-190881855 | Loss | Yes |
| 53 | chr1:191343856-191599062 | Gain | Yes |
| 54 | chr2:6949063-7046220 | Gain | Yes |
| 55 | chr2:7440778-7719067 | Loss | Yes |
| 56 | chr2:9278763-9488806 | Loss | Yes |
| 57 | chr2:11132191-11289889 | Loss | Yes |
| 58 | chr2:15177287-15373324 | Loss | Yes |
| 59 | chr2:19317234-19448666 | Loss | Yes |
| 60 | chr2:21420223-21479637 | Loss | Yes |
| 61 | chr2:24904788-24990847 | Gain | Yes |
| 62 | chr2:26631406-26856762 | Loss | Yes |
| 63 | chr2:27568774-27675976 | Loss | Yes |
| 64 | chr2:30165145-30477779 | Loss | Yes |
| 65 | chr2:30650461-30708806 | Gain | Yes |
| 66 | chr2:31286810-31480324 | Loss | Yes |
| 67 | chr2:41892291-42239873 | Loss | Yes |
| 68 | chr2:48550979-48734488 | Loss and Gain | Yes |
| 69 | chr2:53807784-53876105 | Gain | Yes |
| 70 | chr2:54690427-54830480 | Loss and Gain | Yes |
| 71 | chr2:56863980-56927824 | Loss | Yes |
| 72 | chr2:57933423-57971828 | Loss | Yes |
| 73 | chr2:69907792-70108774 | Loss | Yes |
| 74 | chr2:71140473-71244336 | Loss | Yes |
| 75 | chr2:71719409-71806526 | Loss | Yes |
| 76 | chr2:72262956-73019929 | Loss | Yes |
| 77 | chr2:73289498-74021046 | Loss | Yes |
| 78 | chr2:74660442-74704556 | Loss | Yes |
| 79 | chr2:79485316-79625120 | Loss | Yes |
| 80 | chr2:86749435-86819420 | Loss | Yes |
| 81 | chr2:98567081-98774594 | Gain | Yes |
| 82 | chr2:99818321-100028816 | Gain | Yes |
| 83 | chr2:102211996-102324121 | Loss | Yes |
| 84 | chr2:108117197-108218548 | Loss | Yes |
| 85 | chr2:108946237-109066876 | Gain | Yes |
| 86 | chr2:109902784-110123694 | Loss | Yes |
| 87 | chr2:114299313-114434635 | Loss | Yes |
| 88 | chr2:115898325-116079550 | Loss | Yes |
| 89 | chr2:124061845-124377551 | Loss | Yes |
| 90 | chr2:130549532-130734057 | Loss | Yes |
| 91 | chr2:136763211-136908143 | Loss | Yes |
| 92 | chr2:139440106-139506731 | Gain | Yes |
| 93 | chr2:142843787-142884245 | Gain | Yes |
| 94 | chr2:149276922-149301687 | Loss | Yes |
| 95 | chr2:154617579-154857617 | Gain | Yes |
| 96 | chr3:908224-979201 | Loss | Yes |
| 97 | chr3:2378356-2467682 | Gain | Yes |
| 98 | chr3:5446664-5672522 | Gain | Yes |
| 99 | chr3:15368741-15614917 | Loss | Yes |
| 100 | chr3:21129250-21295188 | Loss | Yes |
| 101 | chr3:21836550-21889819 | Loss | Yes |
| 102 | chr3:42726805-42828566 | Loss | Yes |
| 103 | chr3:45789985-45864412 | Loss | Yes |
| 104 | chr3:46604544-46671598 | Loss | Yes |
| 105 | chr3:49789304-49834267 | Gain | Yes |
| 106 | chr3:52115435-52187613 | Loss | Yes |
| 107 | chr3:57458929-57521095 | Loss | Yes |
| 108 | chr3:65676875-65706900 | Gain | Yes |
| 109 | chr3:69385035-69556711 | Loss | Yes |
| 110 | chr3:71555138-71912236 | Loss and Gain | Yes |
| 111 | chr3:75763183-75868456 | Gain | Yes |
| 112 | chr3:87474148-87529545 | Gain | Yes |
| 113 | chr3:89469726-89623209 | Loss | Yes |
| 114 | chr3:98075926-98239025 | Gain | Yes |
| 115 | chr3:101488239-101517553 | Loss | Yes |
| 116 | chr3:102723065-103006121 | Loss and Gain | Yes |
| 117 | chr3:103032065-103157531 | Gain | Yes |
| 118 | chr3:108288443-108515492 | Loss | Yes |
| 119 | chr4:1395188-1478216 | Loss | Yes |
| 120 | chr4:10512027-10545426 | Gain | Yes |
| 121 | chr4:15393972-15506150 | Loss | Yes |
| 122 | chr4:18775026-18875393 | Gain | Yes |
| 123 | chr4:19122628-19300081 | Loss and Gain | Yes |
| 124 | chr4:22033253-22209947 | Loss | Yes |
| 125 | chr4:25437136-26057981 | Loss and Gain | Yes |
| 126 | chr4:26237323-26315318 | Loss | Yes |
| 127 | chr4:26968180-27018000 | Gain | Yes |
| 128 | chr4:28431161-28762176 | Loss | Yes |
| 129 | chr4:28949724-29138554 | Loss | Yes |
| 130 | chr4:45431378-45572548 | Gain | Yes |
| 131 | chr4:54469478-54716330 | Loss | Yes |
| 132 | chr4:60937897-61217478 | Loss and Gain | Yes |
| 133 | chr4:62643736-62725093 | Loss | Yes |
| 134 | chr4:63185614-63369296 | Gain | Yes |
| 135 | chr4:75423444-75470799 | Loss and Gain | Yes |
| 136 | chr4:81006540-81070974 | Loss | Yes |
| 137 | chr4:87082448-87135703 | Loss | Yes |
| 138 | chr5:11071466-11310951 | Loss | Yes |
| 139 | chr5:12088601-12210681 | Gain | Yes |
| 140 | chr5:21591242-22284563 | Loss | Yes |
| 141 | chr5:33943902-33977691 | Loss | Yes |
| 142 | chr5:47104753-47169889 | Loss | Yes |
| 143 | chr5:51610940-51634087 | Loss | Yes |
| 144 | chr5:56579671-56669082 | Loss | Yes |
| 145 | chr5:59213644-59488578 | Gain | Yes |
| 146 | chr5:62003134-62237436 | Loss and Gain | Yes |
| 147 | chr6:1518218-1592629 | Gain | Yes |
| 148 | chr6:6937167-6979457 | Loss | Yes |
| 149 | chr6:13573742-14067729 | Loss | Yes |
| 150 | chr6:15989371-16146986 | Loss | Yes |
| 151 | chr6:17983333-18224879 | Loss | Yes |
| 152 | chr6:19556040-19673718 | Loss | Yes |
| 153 | chr6:21648841-21685750 | Loss | Yes |
| 154 | chr6:22220989-22416840 | Loss | Yes |
| 155 | chr6:22454713-22496297 | Loss | Yes |
| 156 | chr6:22847247-22911818 | Gain | Yes |
| 157 | chr6:27566215-27709402 | Loss | Yes |
| 158 | chr6:28463317-28654448 | Loss | Yes |
| 159 | chr6:36231847-36248598 | Loss | Yes |
| 160 | chr7:50841-168716 | Gain | Yes |
| 161 | chr7:913973-956038 | Loss | Yes |
| 162 | chr7:3632047-3666402 | Loss | Yes |
| 163 | chr7:6441884-6548203 | Loss | Yes |
| 164 | chr7:10814398-11106458 | Loss | Yes |
| 165 | chr7:11518141-11606754 | Loss | Yes |
| 166 | chr7:13707090-13824805 | Loss | Yes |
| 167 | chr7:20659438-20847263 | Loss | Yes |
| 168 | chr7:28848861-28979274 | Loss | Yes |
| 169 | chr7:29291070-29671771 | Loss | Yes |
| 170 | chr7:34181822-34244599 | Loss | Yes |
| 171 | chr7:37083252-37124566 | Gain | Yes |
| 172 | chr8:5597-96007 | Gain | Yes |
| 173 | chr8:6605406-6678281 | Loss | Yes |
| 174 | chr8:8947944-9343232 | Loss and Gain | Yes |
| 175 | chr8:11740653-11841126 | Gain | Yes |
| 176 | chr8:15429607-15478077 | Gain | Yes |
| 177 | chr8:17952930-17977502 | Loss | Yes |
| 178 | chr8:18207485-18298238 | Loss | Yes |
| 179 | chr8:19048730-19072474 | Loss | Yes |
| 180 | chr8:19873344-19905671 | Loss | Yes |
| 181 | chr8:30569698-30630313 | Loss | Yes |
| 182 | chr9:1797155-1936688 | Gain | Yes |
| 183 | chr9:15270843-15340595 | Loss | Yes |
| 184 | chr9:22373291-22401886 | Loss | Yes |
| 185 | chr9:25178272-25223419 | Loss | Yes |
| 186 | chr9:25481432-25551011 | Gain | Yes |
| 187 | chr10:1166531-1526841 | Loss | Yes |
| 188 | chr10:7102154-7182285 | Loss and Gain | Yes |
| 189 | chr10:7945101-7980528 | Gain | Yes |
| 190 | chr10:9849967-9872674 | Gain | Yes |
| 191 | chr10:9965049-10080899 | Loss and Gain | Yes |
| 192 | chr10:10498692-10607832 | Loss and Gain | Yes |
| 193 | chr10:12148600-12292376 | Loss | Yes |
| 194 | chr10:21398576-21448143 | Gain | Yes |
| 195 | chr10:21808414-22197374 | Loss | Yes |
| 196 | chr11:70391-114720 | Loss and Gain | Yes |
| 197 | chr11:2610515-2673670 | Loss | Yes |
| 198 | chr11:3196613-3453273 | Loss and Gain | Yes |
| 199 | chr11:5956396-6107783 | Loss | Yes |
| 200 | chr11:6463326-6510711 | Loss | Yes |
| 201 | chr11:8353731-8817099 | Loss | Yes |
| 202 | chr11:9967978-10655654 | Loss | Yes |
| 203 | chr11:11191747-11234018 | Loss | Yes |
| 204 | chr11:11571166-11608843 | Loss | Yes |
| 205 | chr11:12800996-12838283 | Loss | Yes |
| 206 | chr11:14029626-14188069 | Loss | Yes |
| 207 | chr11:14976175-15028287 | Loss | Yes |
| 208 | chr12:1112437-1143728 | Gain | Yes |
| 209 | chr12:1776731-1803933 | Loss | Yes |
| 210 | chr12:12755100-12842081 | Gain | Yes |
| 211 | chr12:18216610-18311095 | Loss | Yes |
| 212 | chr12:18996523-19128049 | Loss | Yes |
| 213 | chr12:20428232-20531973 | Loss and Gain | Yes |
| 214 | chr13:1424027-1450274 | Loss | Yes |
| 215 | chr13:3888783-4017468 | Loss | Yes |
| 216 | chr13:4105415-4139583 | Loss | Yes |
| 217 | chr13:5423908-5537294 | Loss | Yes |
| 218 | chr13:7557113-7704321 | Loss | Yes |
| 219 | chr13:7794628-7843175 | Loss | Yes |
| 220 | chr13:17007170-17132427 | Loss | Yes |
| 221 | chr14:35014-107289 | Gain | Yes |
| 222 | chr14:680920-802679 | Loss | Yes |
| 223 | chr14:1158456-1184737 | Loss | Yes |
| 224 | chr14:11197102-11254115 | Loss | Yes |
| 225 | chr14:11836832-11992482 | Loss and Gain | Yes |
| 226 | chr14:14880297-14924988 | Loss | Yes |
| 227 | chr14:15541349-15653126 | Loss and Gain | Yes |
| 228 | chr15:33266-103493 | Gain | Yes |
| 229 | chr15:2545552-2660058 | Loss | Yes |
| 230 | chr15:3629764-3669726 | Loss | Yes |
| 231 | chr15:11251840-11397795 | Loss | Yes |
| 232 | chr17:1192340-1220684 | Loss | Yes |
| 233 | chr17:7681333-7789143 | Gain | Yes |
| 234 | chr17:8574886-8624674 | Gain | Yes |
| 235 | chr17:11092462-11179187 | Loss and Gain | Yes |
| 236 | chr18:240840-560175 | Loss | Yes |
| 237 | chr18:3553719-3578077 | Loss | Yes |
| 238 | chr18:9983890-10015444 | Loss | Yes |
| 239 | chr18:10842098-10895789 | Gain | Yes |
| 240 | chr20:23827-43541 | Gain | Yes |
| 241 | chr20:1317219-1326535 | Loss | Yes |
| 242 | chr20:2454102-2519876 | Gain | Yes |
| 243 | chr20:4117125-4210499 | Gain | Yes |
| 244 | chr21:5357512-5390350 | Loss | Yes |
| 245 | chr21:6820475-6957264 | Gain | Yes |
| 246 | chr22:2769433-2875448 | Gain | Yes |
| 247 | chr23:5969043-6038354 | Loss and Gain | Yes |
| 248 | chr24:6349575-6399526 | Loss | Yes |
| 249 | chr25:22533-56476 | Gain | Yes |
| 250 | chr25:126676-179145 | Gain | Yes |
| 251 | chr25:1014880-1143457 | Gain | Yes |
| 252 | chr25:1333553-1351933 | Loss | No |
| 253 | chr26:311267-433893 | Loss | Yes |
| 254 | chr26:2096975-2288880 | Loss | Yes |
| 255 | chr26:4339426-4384853 | Loss | Yes |
| 256 | chr27:3563-62686 | Loss and Gain | No |
| 257 | chr27:792205-911870 | Gain | No |
| 258 | chr28:909044-915274 | Loss | Yes |
| 259 | chr28:1735926-1799321 | Gain | Yes |
| 260 | chr28:2297848-2424598 | Loss | Yes |
| 261 | chrZ:4570474-4698881 | Gain | No |
| 262 | chrZ:20760318-20796890 | Loss | Yes |
| 263 | chrZ:22064125-22230347 | Loss | Yes |
| 264 | chrZ:30248955-31181093 | Loss and Gain | Yes |
| 265 | chrZ:33168777-33443586 | Loss | Yes |
| 266 | chrZ:45359291-45445413 | Loss | Yes |
| 267 | chrZ:46408596-46459075 | Loss | Yes |
| 268 | chrZ:54829663-55093728 | Gain | Yes |
| 269 | chrZ:56986178-57301771 | Loss | Yes |
| 270 | chrZ:59994286-60259891 | Loss | Yes |
| 271 | chrZ:64935210-65062597 | Loss | Yes |
