# Supplementary material for: Detection of genome-wide copy number variations in two chicken lines divergently selected for abdominal fat content
Source: BMC Genomics. 2014 Jun 24;15:517. doi: 10.1186/1471-2164-15-517 (PMC4092215; doi:10.1186/1471-2164-15-517)
Supplement: Supplementary file 3 — Additional file 3: Table S3: The CNVRs detected in the fat line. (DOC 174 KB) [file 12864_2013_6209_MOESM3_ESM.doc]

Additional file 3: Table S3 The CNVRs detected in the fat line

| NO. | Position | Status | Is verified by CNVPartition |
| --- | --- | --- | --- |
| 1 | chr1:1464907-1566478 | Loss | Yes |
| 2 | chr1:2583691-2689014 | Loss | Yes |
| 3 | chr1:2769500-2900935 | Loss | Yes |
| 4 | chr1:13633539-13877249 | Loss | Yes |
| 5 | chr1:15427537-15511396 | Loss | Yes |
| 6 | chr1:18444585-19186283 | Loss and Gain | Yes |
| 7 | chr1:41181878-41447937 | Loss | Yes |
| 8 | chr1:49654223-49699886 | Gain | Yes |
| 9 | chr1:50632934-50714720 | Loss | Yes |
| 10 | chr1:64792036-64832004 | Loss | Yes |
| 11 | chr1:67413843-67488278 | Loss | Yes |
| 12 | chr1:68169551-68304034 | Loss | Yes |
| 13 | chr1:71729385-71789895 | Loss | Yes |
| 14 | chr1:72807948-72899839 | Loss | Yes |
| 15 | chr1:73452365-73632233 | Gain | Yes |
| 16 | chr1:96528587-96573079 | Loss | Yes |
| 17 | chr1:100948030-100994703 | Loss | Yes |
| 18 | chr1:105458422-105534119 | Loss | Yes |
| 19 | chr1:106899649-107117673 | Loss | Yes |
| 20 | chr1:131493260-131555066 | Loss | Yes |
| 21 | chr1:132802127-132885799 | Loss | Yes |
| 22 | chr1:138559515-138734596 | Loss | Yes |
| 23 | chr1:150876580-151225096 | Loss | Yes |
| 24 | chr1:151798977-152400450 | Loss | Yes |
| 25 | chr1:152688614-153253682 | Loss | Yes |
| 26 | chr1:154589974-154622574 | Loss | Yes |
| 27 | chr1:154879486-155158254 | Loss | Yes |
| 28 | chr1:156390993-156511670 | Loss | Yes |
| 29 | chr1:157298758-157389883 | Loss | Yes |
| 30 | chr1:163750248-164505622 | Loss | Yes |
| 31 | chr1:169521537-169624958 | Loss | Yes |
| 32 | chr1:179442538-179486349 | Gain | Yes |
| 33 | chr1:181893231-181993951 | Loss | Yes |
| 34 | chr1:183081919-183191352 | Loss | Yes |
| 35 | chr1:185222656-185315571 | Loss | Yes |
| 36 | chr2:2981117-3166350 | Loss | Yes |
| 37 | chr2:3658541-3739002 | Loss | Yes |
| 38 | chr2:5346033-5593287 | Loss | Yes |
| 39 | chr2:8082563-8151102 | Loss | Yes |
| 40 | chr2:11806317-12540390 | Loss | Yes |
| 41 | chr2:18269812-18962790 | Loss and Gain | Yes |
| 42 | chr2:23409107-23447146 | Loss | Yes |
| 43 | chr2:24584605-24990847 | Loss | Yes |
| 44 | chr2:25584309-25622388 | Loss | Yes |
| 45 | chr2:26079974-26111296 | Gain | Yes |
| 46 | chr2:28578820-28706749 | Loss | Yes |
| 47 | chr2:28904806-29071991 | Loss | Yes |
| 48 | chr2:40966378-41080531 | Loss | Yes |
| 49 | chr2:48582555-48667693 | Loss | Yes |
| 50 | chr2:53807784-53903578 | Loss | Yes |
| 51 | chr2:54712053-54812923 | Loss | Yes |
| 52 | chr2:62053255-62149156 | Loss | Yes |
| 53 | chr2:65211891-65271388 | Loss | Yes |
| 54 | chr2:89608780-89814926 | Loss | Yes |
| 55 | chr2:123169926-123198312 | Loss | Yes |
| 56 | chr2:124150699-124821424 | Loss | Yes |
| 57 | chr2:133793413-133900730 | Loss | Yes |
| 58 | chr2:137808929-137874455 | Loss | Yes |
| 59 | chr2:139954838-140199371 | Loss | Yes |
| 60 | chr2:147326101-147576361 | Loss | Yes |
| 61 | chr3:33720566-33762789 | Gain | Yes |
| 62 | chr3:43548451-43585451 | Loss | Yes |
| 63 | chr3:46327258-46386338 | Loss | Yes |
| 64 | chr3:46604544-46701497 | Loss | Yes |
| 65 | chr3:46739299-46894163 | Loss | Yes |
| 66 | chr3:49355005-49546491 | Loss | Yes |
| 67 | chr3:55644653-55671656 | Loss | Yes |
| 68 | chr3:57458929-57546034 | Loss | Yes |
| 69 | chr3:58727667-58801687 | Loss | Yes |
| 70 | chr3:62790524-62858153 | Loss | Yes |
| 71 | chr3:69478370-69556711 | Loss | Yes |
| 72 | chr3:69906698-70119483 | Loss | Yes |
| 73 | chr3:72001664-72186394 | Loss and Gain | Yes |
| 74 | chr3:82621091-82782647 | Loss | Yes |
| 75 | chr3:88044533-88188780 | Loss | Yes |
| 76 | chr3:93411484-93477157 | Loss | Yes |
| 77 | chr3:98135241-98239025 | Loss and Gain | Yes |
| 78 | chr3:102920029-103138310 | Loss | Yes |
| 79 | chr3:105449520-105483561 | Loss | Yes |
| 80 | chr4:15987949-16031234 | Gain | Yes |
| 81 | chr4:18727558-19300081 | Loss and Gain | Yes |
| 82 | chr4:22055572-22101523 | Loss | Yes |
| 83 | chr4:23520111-23564682 | Loss | Yes |
| 84 | chr4:23953310-24205318 | Loss | Yes |
| 85 | chr4:24323786-24409089 | Loss and Gain | Yes |
| 86 | chr4:25792386-26085937 | Loss | Yes |
| 87 | chr4:44431071-44573478 | Loss | Yes |
| 88 | chr4:57366708-57498005 | Loss | Yes |
| 89 | chr4:58075092-58137944 | Loss | Yes |
| 90 | chr4:60832038-61254575 | Loss | Yes |
| 91 | chr4:61693514-61768067 | Loss | Yes |
| 92 | chr4:63334389-63481290 | Loss | Yes |
| 93 | chr4:63953044-64066228 | Loss | Yes |
| 94 | chr4:64259257-64346450 | Loss | Yes |
| 95 | chr4:64645582-64915020 | Loss | Yes |
| 96 | chr4:65513802-66055249 | Loss | Yes |
| 97 | chr4:66770452-66870266 | Loss | Yes |
| 98 | chr4:69473528-69588750 | Loss | Yes |
| 99 | chr4:72018808-72122237 | Loss | Yes |
| 100 | chr4:72903667-74346663 | Loss and Gain | Yes |
| 101 | chr4:84476641-84521727 | Loss | Yes |
| 102 | chr4:84688123-84746294 | Gain | Yes |
| 103 | chr5:12088601-12756196 | Loss and Gain | Yes |
| 104 | chr5:14445307-14480770 | Loss | Yes |
| 105 | chr5:29758734-29893032 | Loss | Yes |
| 106 | chr5:32366746-32535159 | Loss | Yes |
| 107 | chr5:33373065-33628432 | Loss and Gain | Yes |
| 108 | chr5:35954456-36426195 | Loss | Yes |
| 109 | chr5:39077662-39524853 | Loss | Yes |
| 110 | chr5:44155259-44778729 | Loss | Yes |
| 111 | chr5:51940474-51976354 | Loss | Yes |
| 112 | chr5:59811459-60007708 | Loss | Yes |
| 113 | chr6:4044641-4108152 | Loss and Gain | Yes |
| 114 | chr6:17959771-18026636 | Loss | Yes |
| 115 | chr6:19470652-19595324 | Loss | Yes |
| 116 | chr6:20576630-20645020 | Loss | Yes |
| 117 | chr6:22454713-22676219 | Loss | Yes |
| 118 | chr6:22828584-23201253 | Loss | Yes |
| 119 | chr6:25535062-25560787 | Gain | Yes |
| 120 | chr6:33063385-33097841 | Gain | Yes |
| 121 | chr7:9547479-9746560 | Loss | Yes |
| 122 | chr7:10360081-10456453 | Loss | Yes |
| 123 | chr7:16633154-16740098 | Gain | Yes |
| 124 | chr7:33144407-33275407 | Loss | Yes |
| 125 | chr8:5597-651415 | Loss | Yes |
| 126 | chr8:9249489-9395195 | Loss | Yes |
| 127 | chr8:9931093-10016422 | Loss | Yes |
| 128 | chr8:12167400-12725373 | Loss | Yes |
| 129 | chr8:14008179-14211497 | Loss and Gain | Yes |
| 130 | chr8:18957245-19072474 | Loss | Yes |
| 131 | chr8:21877314-21925081 | Loss | Yes |
| 132 | chr9:7219744-7274029 | Loss | Yes |
| 133 | chr9:12839846-12921387 | Loss and Gain | Yes |
| 134 | chr9:13171628-13228069 | Loss | Yes |
| 135 | chr9:22638514-22791132 | Loss | Yes |
| 136 | chr9:23496401-23551071 | Gain | Yes |
| 137 | chr9:23884611-23975936 | Gain | Yes |
| 138 | chr9:25195829-25551011 | Loss and Gain | Yes |
| 139 | chr10:4405328-4509030 | Loss | Yes |
| 140 | chr10:6806958-6982589 | Loss | Yes |
| 141 | chr10:7244604-7266256 | Loss | Yes |
| 142 | chr10:7435039-7462870 | Loss | Yes |
| 143 | chr10:8635259-8671067 | Gain | Yes |
| 144 | chr10:9367350-9688108 | Loss and Gain | Yes |
| 145 | chr10:10479815-10573304 | Loss | Yes |
| 146 | chr10:10607832-10690471 | Loss and Gain | Yes |
| 147 | chr10:12898115-13008498 | Loss | Yes |
| 148 | chr10:17778463-17815970 | Loss | Yes |
| 149 | chr10:21381753-21448143 | Loss | Yes |
| 150 | chr10:21991204-22197374 | Loss | Yes |
| 151 | chr11:59939-114720 | Loss | Yes |
| 152 | chr11:3196613-3352571 | Loss and Gain | Yes |
| 153 | chr11:13655272-14349058 | Loss | Yes |
| 154 | chr11:14819127-14934095 | Loss | Yes |
| 155 | chr11:19352318-19384494 | Loss | Yes |
| 156 | chr12:12697362-12842081 | Gain | Yes |
| 157 | chr12:13729306-13843030 | Loss | Yes |
| 158 | chr12:15104701-15152357 | Loss | Yes |
| 159 | chr13:1344556-1533552 | Loss | Yes |
| 160 | chr13:10535130-10551597 | Gain | Yes |
| 161 | chr13:10923389-10936549 | Gain | Yes |
| 162 | chr14:2511980-2538380 | Gain | Yes |
| 163 | chr14:7492472-7511980 | Loss | Yes |
| 164 | chr14:12138193-12178126 | Gain | Yes |
| 165 | chr17:6742435-6827479 | Loss | Yes |
| 166 | chr17:7948097-7984218 | Loss | Yes |
| 167 | chr17:9223286-9247986 | Gain | Yes |
| 168 | chr17:11092462-11179187 | Loss and Gain | Yes |
| 169 | chr18:365479-406185 | Loss | Yes |
| 170 | chr18:456791-482375 | Gain | Yes |
| 171 | chr21:6833550-6886552 | Loss | Yes |
| 172 | chr22:2809920-2875448 | Loss | Yes |
| 173 | chr22:3892168-3934387 | Loss | Yes |
| 174 | chr25:683-152000 | Loss and Gain | No |
| 175 | chr26:3769261-3812416 | Loss | Yes |
| 176 | chr27:3563-24802 | Loss and Gain | No |
| 177 | chr27:1321674-1339777 | Loss | No |
| 178 | chr28:1735926-1736259 | Loss | Yes |
| 179 | chr28:2247072-2256352 | Gain | Yes |
| 180 | chrZ:9933957-10124452 | Gain | Yes |
| 181 | chrZ:22264176-22387697 | Loss | Yes |
| 182 | chrZ:30475720-30856682 | Loss and Gain | Yes |
| 183 | chrZ:31181093-31296039 | Loss | Yes |
| 184 | chrZ:38926467-39031651 | Loss | Yes |
| 185 | chrZ:50902469-51027069 | Loss | Yes |
| 186 | chrZ:62739744-62787579 | Gain | Yes |
| 187 | chrZ:62995000-63110556 | Gain | Yes |
| 188 | chrZ:72011220-72054550 | Gain | Yes |
